# Impact of age at first sexual intercourse on the development and prognosis of breast cancer: A two-sample Mendelian randomization study with NHANES validation

**DOI:** 10.1097/MD.0000000000043676

**Published:** 2025-08-01

**Authors:** Xinghuang Yang, Tianyu Deng, Tianai Xu, Min Ma

**Affiliations:** a Yangzhou University Medical College, Yangzhou University, Yangzhou, Jiangsu Province, China; b Department of Obstetrics and Gynecology, Affiliated Hospital of Yangzhou University, Yangzhou University, Yangzhou, Jiangsu Province, China; c Jiangsu Key Laboratory of Non Coding RNA Basic and Clinical Transformation, Yangzhou University, Yangzhou, Jiangsu Province, China.

**Keywords:** breast cancer, female reproduction, first sexual intercourse, Mendelian randomization, NHANES

## Abstract

This study examines the potential link of age at first sexual intercourse (AFS) with breast cancer (BC). A two-sample Mendelian randomization (MR) method was leveraged to examine the possible link of AFS with BC. The causal effect of AFS on BC was primarily appraised by an inverse variance weighted method. Sensitivity analyses were applied to appraise the stability of MR results, involving Cochran Q test, MR-Egger intercept analysis, outlier test (MR-pleiotropy residual sum and outlier), and leave-one-out method. The National Health and Nutrition Examination Survey was leveraged to validate the impact of AFS on BC prognosis. Kaplan–Meier curves, Cox regression analysis, and restricted cubic splines were generated to appraise the link of AFS with BC. The inverse variance weighted exhibited a positive causal effect of AFS on BC (odds ratio = 1.237, 95% confidence interval = 1.077–1.420, *P* = .003) with reliable and stable results. Nevertheless, other methods revealed no notable association. In addition, none of the above sensitivity analyses revealed any violation of the MR assumptions. The results from the investigated sample cohort of 92,062 women showed that a high AFS was tied to worse outcomes in BC patients (hazard ratio = 1.148, 95% confidence interval = 1.048–1.258, *P* = .003). This correlation remained consistent across various subgroups. The study suggests that there is a noticeable link between AFS and BC, providing further evidence for predicting the risk and prognosis of BC.

## 1. Background

Breast cancer (BC) is the most prevalent malignant tumor in women. It represents the primary cause of cancer-related mortality among women. According to the latest statistics in 2022, BC is the second most frequently diagnosed and the fourth leading cause of cancer-related mortality globally, making it the highest in both incidence and mortality among cancers affecting women.^[1]^ Due to large individual differences in the timing of onset, growth rate, and risk of metastasis, BC screening varies for different ethnic and geographic populations. The U.S. Preventive Services Task Force recently updated recommendations for BC screening,^[2]^ highlighting the importance of initiating BC screening at an earlier age for women. Although many identified factors can predict the occurrence of BC, most of them are limited in accuracy, potentially leading to missed diagnosis or over-screening. Hence, it is essential to identify more reliable risk factors for early-stage BC to reduce the incidence of BC in the population.

The causes of BC are complex and probably be linked to age, genetics, life, environment, endocrine hormone levels, and other factors. These factors rarely affect BC in isolation. Instead, they work collectively to contribute to BC. Recently, the pathogenetic role of reproductive behaviors (like age at menarche, age at first sexual intercourse [AFS], age at primigravida, and age at menopause) in the natural history of BC has received increasing attention.^[3]^ The link of each reproductive behavior with BC risk varies according to different molecular subtypes of BC. For example, later age at menarche and breastfeeding is tied to a reduced BC risk for all subtypes, whereas later age at menopause and primigravida is linked to an increased BC risk for only some subtypes (tubular A, tubular B, and HER2).^[4]^ However, the extent to which reproductive factors affect BC remains unclear due to the inherent statistical errors, which cannot be eliminated. Therefore, there is a real need to refine the mechanisms and relationships that may exist between the two.

Recently, multiple studies have begun to investigate the relevance of AFS to vital health. These studies have demonstrated that AFS possesses considerable research value in cancer. Genetically, single nucleotide polymorphisms (SNPs) in AFS have been recognized and found to have an impact on health and evolutionary fitness.^[5]^ Disease-wise, AFS has been verified to be causally linked to the risks of cardiovascular diseases^[6]^ and major depressive disorder.^[7]^ As an important component of reproductive factors, AFS might be associated with BC risk. In contrast, the link of AFS with BC has garnered less attention. On the one hand, it concerns privacy, leading to limited or inaccurate access to AFS data. On the other hand, AFS is essentially a point in time and not indicative of the process. However, it is very important and signals a huge potential for changes in reproductive factors that could impact BC. One Mendelian randomization (MR) study exploring the effects of AFS on reproductive and behavioral outcomes reveals that AFS is indeed linked to several life history outcomes. However, evidence of horizontal pleiotropy violated MR assumptions, and thus causality could not be accurately inferred.^[8]^

MR is a novel method for revealing cancer. It has been widely employed in identifying BC target genes and screening corresponding targeted drugs.^[9,10]^ Nonetheless, although MR has been employed to study the link between AFS and BC,^[11]^ insufficient validated evidence is available to elucidate the link. Therefore, this study leverages a two-sample MR method to examine the possible link of AFS with BC risk based on a publicly available database of large-sample genome-wide association studies (GWAS), aiming to enhance early screening and prevention of BC. In addition, on the basis of the MR results, the National Health and Nutrition Examination Survey (NHANES) is leveraged to verify the impact of AFS on BC prognosis and survival duration.

## 2. Methods

### 2.1. Mendelian randomization

#### 2.1.1. Study design

**Figure 1.**
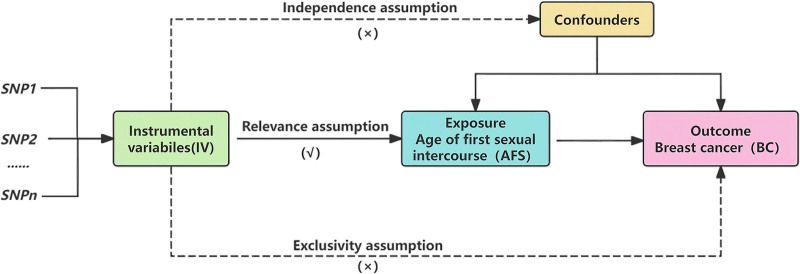
Mendelian randomization research design.

This study leveraged two-sample MR to assess the causal link of AFS with BC risk. Three assumptions needed to be fulfilled for MR analysis: (i) association assumption, (ii) independence assumption, and (iii) exclusivity assumption.^[12]^ Based on the 3 assumptions, data from genetic genes were utilized as instrumental variables (IVs) (Fig. 1). The specific process was as follows: firstly, genetic variants strongly tied to AFS at the genome-wide significance level were selected to fulfill the association assumption. Secondly, the independence assumption was ensured by verifying that the genetic variants were not connected to other potential factors tied to AFS and BC. Finally, the exclusivity assumption was ensured by applying a genome-wide significance threshold and specifying that genetic variants were only associated with BC except for AFS.

#### 2.1.2. Data sources

GWAS data for AFS and BC were obtained from https://gwas.mrcieu.ac.uk/. Exposure data related to AFS were extracted from a 2021 GWAS study hosted by Mills MC, including a sample size of 397,338 cases from European populations and 16,359,424 SNPs identified. Outcome data tied to BC were extracted from a 2021 GWAS study hosted by Sakaue S, which included a sample size of 257,730 cases (a European pedigree of 17,389 cases and 240,341 controls) and identified 24,133,589 SNPs. Among these, the sex, consortium, subcategory/category, and ontology of the 2 data sets were unknown. However, they were all constructed from the human genome version HG19/GRCh37 (Table S1, Supplemental Digital Content, https://links.lww.com/MD/P611).

#### 2.1.3. IV selection

The MR study used SNPs as IVs and screened for SNPs notably tied to AFS across the genome using the *P* < 5 × 10^‐8^ criterion.^[13,14]^ The clumping operation was performed using the criterion of *R*^2^ < 0.001 and window size = 10,000kb to remove linkage disequilibrium between SNPs,^[15]^ thus reducing the random frequency of co-inheritance of some alleles at different gene positions and attenuating the interference with the results. The allelic orientations of the SNPs in the exposure data and the outcome data were synergized by the Harmonization operation. SNPs with palindromic sequences or incompatible SNPs were eliminated, thus improving the accuracy and reliability of the subsequent MR analysis. To ensure that the chosen SNPs for AFS were closely connected to BC risk and to avoid potential weak instrumental bias, this study assessed the strength of IV by calculating the *F* value of each SNP. *F* > 10 indicated no weak instrumental bias.^[16]^

#### 2.1.4. MR analysis

Inverse variance weighted (IVW) was primarily leveraged for calculating odds ratio (ORs) and 95% confidence intervals (CIs).^[17]^ IVW requires that genetic variants can only impact the outcome through the exposures, thereby making causal relationships highly detectable. Although SNPs strongly linked to outcome, along with some palindromic sequences and incompatible SNPs, have been largely removed, it cannot be entirely ruled out that other unknown confounders may cause genetic pleiotropy, potentially impacting the results. Thus, MR Egger, weighted median, simple mode, weighted mode, and other analysis methods were also employed. If all the above 5 methods yielded similar results for causal inference, the results could be considered reliable. If only the IVW method or some of the methods presented positive results, the results could not be fully considered reliable. This suggested that AFS might also be influenced by other established factors, which may work together to contribute to the occurrence of BC.

#### 2.1.5. Sensitivity analysis

The heterogeneity test was employed to examine the degree of difference between the BC group and the normal group. In this study, Cochran Q test was employed to detect heterogeneity among SNPs.^[18]^ If *P* > .05, it meant no marked heterogeneity, in which case a fixed-effects model was employed. Conversely, a random-effects model was leveraged, and heterogeneity did not affect the reliability of the results. Considering the potential influence of genetic variation on causal associations, this study mainly used the MR-Egger intercept method to jointly test for horizontal pleiotropy.^[19]^ If the intercept was not equal to 0, it meant notable horizontal pleiotropy. This implied that the outcome might exist prior to any exposure factor, thereby rendering the study meaningless. On the contrary, both the research process and the results were meaningful. Finally, leave-one-out and MR-pleiotropy residual sum and outlier were used to determine the influence of individual SNPs on causality and assess the stability of MR analysis results.^[20]^ If outliers (SNPs with *P* < .05) were present, causal associations would be reestimated after removing them to rectify horizontal pleiotropy.

### 2.2. National Health and Nutrition Examination Survey

#### 2.2.1. Study population

**Figure 2.**
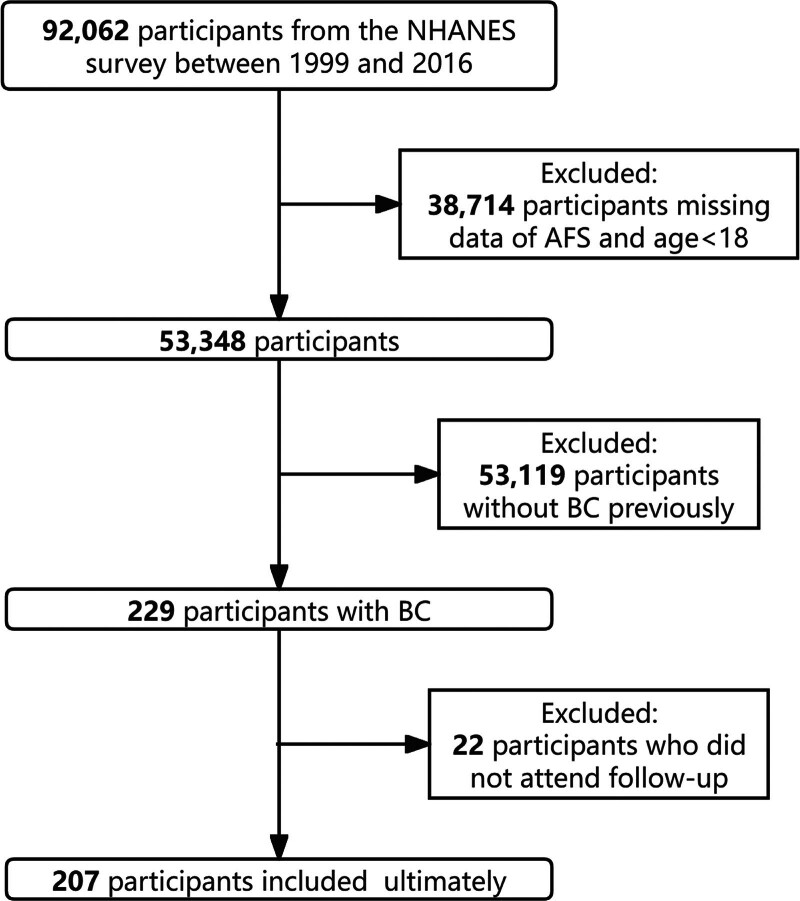
NHANES data selection flow chart. NHANES = National Health and Nutrition Examination Survey.

NHANES is a national survey conducted by the National Center for Health Statistics to appraise the health and nutrition status of Americans.^[21]^ The study used BC samples collected from the 1999 to 2016 NHANES survey (http://www.cdc.gov/nchs/nhanes/) to uncover the association between AFS and BC prognosis. A total of 92,062 individuals participated in the survey. Only 207 individuals with BC were included in this study (Fig. 2).

#### 2.2.2. NHANES analysis

Cox proportional hazard models were leveraged to appraise the link of AFS with all-cause mortality (ACM) among BC participants. Covariates were adjusted in 3 models. Specifically, no adjustments were conducted in Model 1. Moreover, simple adjustments for age, race, poverty-to-income ratio (PIR), and education level were made in Model 2. Furthermore, on the basis of Model 2, additional adjustments for body mass index (BMI), smoking status, drinking status, hypertension, and diabetes were made in Model 3 (Table 1). Kaplan–Meier curves were leveraged to display the censored data and the different survival patterns for BC participants at various follow-up times. Multiple imputation methods were applied to address the missing data. Restricted cubic splines (RCS) were used to appraise the nonlinear link of AFS with ACM in BC participants. Knots for the RCS curves were chosen based on the minimization of Akaike Information Criterion. Moreover, stratified analysis and interaction tests were used to examine the link of AFS with ACM in several subgroups based on race, hypertension, and diabetes.

**Table 1:** Association between AFS and all-cause mortality of patients with BC based on multivariable cox proportional hazard models.

	HR (95% CI)	*P*
Model 1	1.072 (0.994–1.155)	.071
Model 2	1.086 (1.002–1.177)	.043
Model 3	1.148 (1.048–1.258)	.003

*Notes*: Model 1: no covariates adjusted. Model 2: adjusted for age, race, PIR, and education level. Model 3: includes all adjustments from Model 2, with additional adjustments for BMI, smoking status, drinking status, hypertension, and diabetes.

AFS = age at first sexual intercourse, BC = breast cancer, BMI = body mass index, CI = confidence interval, HR = hazard ratios, PIR = poverty-to-income ratio.

### 2.3. Statistical analysis

MR analyses were implemented by the R package “Two Sample MR” (Version 0.6.6) in R (Version 4.3.3) and NHANES analyses were executed by means of the R (Version 4.3.3). ORs and hazard ratios (HRs) with corresponding 95% CIs were computed. A difference of *P* < .05 between the 2 sides was considered statistically significant. The results of the study were visualized by generating MR scatter plots, forest plots, funnel plots, and leave-one-out images.

## 3. Results

### 3.1. MR results

#### 3.1.1. IV selection

A total of 196 SNPs were included after removing 38 SNPs with palindromic sequences and one incompatible SNP (rs62177795) (Table S2, Supplemental Digital Content, https://links.lww.com/MD/P611). A formula $F=\frac{N-K-1}{K}\times \frac{R^{2}}{1-R^{2}}$ was employed to measure the validity of the selected SNPs as IVs.^[22]^ Among this formula, N was the sample size in the exposure database, K was the number of SNPs included, and *R*^2^ was the proportion of exposure variants in the exposure database explained by SNPs. Moreover, another formula $R^{2}=2\times (1-MAF)\times MAF\times beta^{2}$ was utilized to compute the strength of the association between SNP and exposure.^[23]^ Minor allele frequency (MAF) represented the minimum allele frequency and beta was a metric to quantify the strength of the association between exposure and outcome. The absence of effect allele frequency (Eaf) values in the exposure data on AFS in the original GWAS summary data prevented the calculation of MAF values (if Eaf > 0.5, MAF = 1‐Eaf; if Eaf < 0.5, MAF = Eaf). Therefore, a traditional formula, $F=\beta \times \frac{{beta}^{2}}{se^{2}}$,^[24]^ was applied to calculate the *F* value of each exposure SNP separately. Finally, the average value was taken, resulting in an average *F* value of 45.25 (>10) (the maximum value was 171.49, and the minimum value was 27.44). This indicated that all the SNPs were strongly linked to the risk of BC. Therefore, there were no weak IVs to produce bias.

#### 3.1.2. Two-sample MR analysis

**Figure 3.**
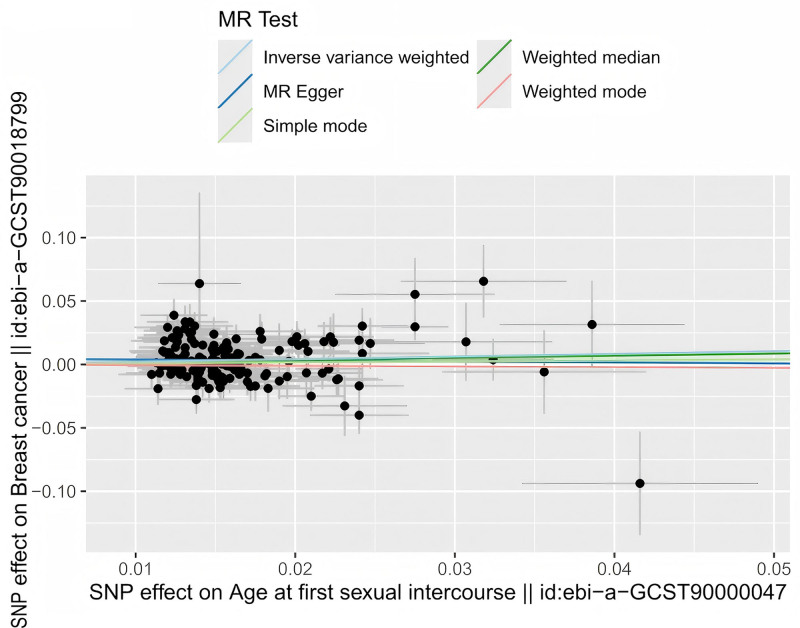
Scatter plot for the causal association between AFS and BC. AFS = age at first sexual intercourse, BC = breast cancer.

In this study, the MR analysis was implemented using 5 methods: MR Egger, weighted median, IVW, simple mode, and weighted mode. Among them, the results of IVW genetic prediction showed a causal link between AFS and BC (beta = 0.2124, SE = 0.0707, *P* = .0027). Moreover, AFS was positively connected to BC risk. Individuals with later AFS had a 1.24 times higher risk of BC than normal individuals (OR = 1.2366, 95%CI = 1.0766–1.4205). However, no causal association was found between AFS and BC by any of the other 4 methods (all *P* > .05) (Table S3, Supplemental Digital Content, https://links.lww.com/MD/P611). The IVW method demonstrated a positive link between AFS and BC, indicating that later AFS increased the risk of BC (Fig. 3). The correlation direction from the weighted median and simple mode methods aligned with that derived from the IVW method, whereas the MR Egger and weighted mode methods revealed an opposite direction (Fig. 3). The impact of SNP on BC risks is illustrated in the forest plot. The IVW method demonstrated that the risk of BC rose with increasing AFS, opposing the results of the MR Egger method (Fig. S1, Supplemental Digital Content, https://links.lww.com/MD/P610).

#### 3.1.3. Sensitivity analysis

The results of MR-Egger regression analyses showed that the intercept was almost equal to 0 (Egger_intercept = 0.0046, *P* = .3404), suggesting no horizontal pleiotropy and confirming the plausibility and reliability of the results (Fig. 3). Both the MR-Egger method (*P* = .0014) and IVW method (*P* = .0014) indicated heterogeneity between the IVs of AFS used in the MR analysis. Hence, a random-effects model was chosen (Table S4, Supplemental Digital Content, https://links.lww.com/MD/P611, Fig. S2, Supplemental Digital Content, https://links.lww.com/MD/P610). MR analyses were performed again and yielded the following results: IVW (*P* = .0029), MR Egger (*P* = .8090), weighted median (*P* = .0691), simple mode (*P* = .7846), weighted mode (*P* = .8553). The MR-pleiotropy residual sum and outlier was firstly detected for original exposure data (SD = 0.2124, T-stat = 0.0707, *P* = .0031), followed by global test (RSSObs = 218.2041, *P* = .0016) and outlier test (P: rs1609598 = 0.8848; rs4961705 = 0.9796; rs11240331 = 0.3476, and *P* > 1 for the rest of the SNPs). No notably abnormal outliers were observed (Table S4, Supplemental Digital Content, https://links.lww.com/MD/P611, Fig. S2, Supplemental Digital Content, https://links.lww.com/MD/P610). The results of the leave-one-out analysis indicated that after eliminating each SNP in turn and calculating the meta-effects of the remaining SNPs, all the error lines were found to be on the right side of 0, indicating that the results were also reliable (Fig. S3, Supplemental Digital Content, https://links.lww.com/MD/P610).

### 3.2. National Health and Nutrition Examination Survey

#### 3.2.1. Baseline characteristics

From 1999 to 2016, 92,062 participants were included in NHANES. Among these, 207 individuals had a history of BC. The median age was 56.38 years and the median AFS was 18.91 years. All the participants were female. Most participants were non-Hispanic white (97 cases, 46.86%). Over 65% of the participants had a college education or above. Nearly 50% of the participants had a BMI >30. 57.49% of the participants never smoked and 72.95% never drank. 57.97% had hypertension and 32% had diabetes. The median eGFR was 89.96 mL/min/1.73 m^2^. The median follow-up time was 9.12 years. A total of 184 cases of survival and BC-related mortality were observed. Compared with survivors, non-survivors presented low educational levels and PIR (*P* < .05). In addition, other characteristics did not notably affect the survival of BC patients due to the small sample size (Table S5, Supplemental Digital Content, https://links.lww.com/MD/P611).

#### 3.2.2. Association between AFS and BC

The Cox regression analysis illustrated that a certain extent value of AFS was linked to worse outcomes of BC patients, although the effect was weak. It was observed that each incremental unit of AFS was remarkably connected to a 15% elevated likelihood of developing BC (HR = 1.148, 95%CI = 1.048–1.258, *P* = .003) (Table 1). The Kaplan–Meier curve also showed that the survival probability trends for BC patients varied with follow-up time between those under <18 years old and those over 18, though the results were less credible due to the small sample size (Log-rank *P* = .93) (Fig. S4, Supplemental Digital Content, https://links.lww.com/MD/P610). The multivariate-adjusted RCS curve revealed a nonlinear link between AFS and ACM in BC participants (*P* for nonlinear = .0302 < .05). The knot was located at 19, and the HR monotonically decreased prior to this point and increased thereafter (Fig. S5, Supplemental Digital Content, https://links.lww.com/MD/P610).

#### 3.2.3. Subgroup analysis

Considering potential risk factors, a subgroup analysis was performed to identify interaction effects by stratifying the data by race, hypertension, diabetes, and AFS (Table S6, Supplemental Digital Content, https://links.lww.com/MD/P611). Notable interaction effects were noted in the non-Hispanic white (HR = 1.27, 95% CI = 1.17–1.379, *P* < .001), no-hypertension (HR = 1.203, 95% CI = 1.104–1.311, *P* < .001), and AFS (age ≥ 18) (HR = 1.175, 95% CI = 1.079–1.281, *P* < .001) groups, aligning with previous results.

## 4. Discussion

This study integrates GWAS data from public databases, using a two-sample MR experimental design to establish a noticeable link between AFS and the risk of BC. The result suggests that later AFS is tied to a risen risk of BC. In addition, we employ the data from the NHANES database to validate the crucial role of AFS in BC prognosis, confirming MR results and offering strong evidence and novel insights for future clinical work.

The onset of BC is associated with a number of specific genetic variants, like BRCA1/2, HER-2, TP53, PTEN, ATM, CDH1, and RAD51C/D. Several studies have investigated BRCA1/2. They employed diverse biotechnological tools to induce mutations and observe survival in different clinical settings, so as to intervene in the progression and prognosis of all stages of BC.^[25–27]^ The NCCN Clinical Practice Oncology has assessed genetic and familial high risks for BC and suggests genetic testing and guidance for individuals with pathogenic variants.^[28]^ Interestingly, AFS has also been shown to be genetically related. This is consistent with our MR results. A previous study has identified 38 loci associated with AFS and hypothesized that early puberty may advance sexual debut and lead to diseases linked to reproductive aspects.^[29]^ Moreover, some investigators have integrated the AFS in 387,338 cases and identified 282 associated SNPs. Notably, several of these SNPs are linked to key genes related to follicle-stimulating hormone (FSH). They conclude that individuals with higher polygenic scores tend to have their first sexual intercourse later.^[5]^ It is not clear whether there is any crossover between the causative genes for BC and the anchor genes for AFS. However, given the results of the current MR study, it is reasonable to hypothesize that certain genes may influence individuals to delay the occurrence of first sexual intercourse while facilitating the onset of BC at the same time.

The factors contributing to the increased risk of BC linked to AFS are multifaceted. Numerous studies have revealed that the onset of BC is closely linked to the hormone levels in the female organism, including estrogen, progesterone, FSH, and luteinizing hormone, which can be carcinogenic by promoting the abnormal proliferation of breast tissue. For example, a meta-analysis has shown that menopausal hormone therapy can increase the incidence of BC, with the highest risk observed in those receiving estrogen combined with progestin.^[30]^ Moreover, Peter J. Park et al have identified a common pattern of oncogene amplification through DNA sequencing and data analysis, and they propose estrogen as a mechanistic source of BC.^[31]^ FSH promotes estrogen secretion, which in turn promotes endometrial hyperplasia in preparation for conception. In recent years, an increasing body of evidence supports that high levels of postmenopausal FSH and luteinizing hormone are risk factors for BC.^[32]^ It can be hypothesized that the expression of FSH-related genes in AFS indirectly promotes estrogen secretion by increasing FSH levels, thus leading to the development of BC.

In addition, a delay in AFS itself may lead to late marriage and late childbearing in women, and advanced maternal age is also a high-risk factor for BC. Although breastfeeding has a better protective effect against BC, the rapid degradation of the mammary glands after returning to breastfeeding in advanced maternal age may promote the occurrence, growth, and metastasis of BC.^[33,34]^

Interestingly, later AFS due to pathologic phenomena may also lead to the development of BC. Based on the identified genetic loci for AFS,^[29]^ a study has found that the advancement of puberty can increase the risk of BC in the human body.^[35]^ However, AFS, as a reproductive behavior, is influenced by both the external environment and genetic factors. It is also related to social phenomena like the level of education, socio-economic status, and state of mind,^[5]^ which are consistent with the NHANES analysis results. Therefore, more research needs to be done in the future to explore the complex relationship.

The study offers certain strengths compared to other studies. The principle of MR utilizes the first and second Mendelian laws: the law of segregation and the law of independent assortment.^[36]^ The most notable feature of MR is that genetic variation is randomly assigned to the offspring during the process of gamete formation at the time of conception, and the process is virtually unaffected by life, environmental, and psychological factors. Compared to observational studies, using genetic variation as a proxy for AFS reduces the interference of reverse causal associations and the bias of confounding factors on the results of the study.^[37]^ Moreover, compared with randomized controlled trials, the use of GWAS large-sample statistics can circumvent the inconvenience caused by long-term follow-up of BC.^[38]^ Furthermore, the risk estimated by the MR is a lifetime risk, which has a longer follow-up than other studies. In addition, these results are validated based on the NHANES database and are proven to be complete and credible.

Although this study reveals the relationship between AFS and BC risk, there are still some limitations. (i) All GWAS data are derived from European populations, and further research is needed to determine whether the results are applicable to other populations. (ii) Sex or male/female ratio is not described in the extracted exposure data. Although the probability of BC in men is much lower than that in women, accounting for only 1% of all BCs, sex specificity should not be ignored in exploring the pathogenesis of BC.^[39]^ (iii) The subtypes or grading of BC are not specified in the extracted outcome data, and the magnitude of the effect of AFS on various BCs may differ. The potential mechanisms between AFS and BC need to be explored more deeply in the future.

## 5. Conclusion

This study reveals a potential relationship between AFS and BC by applying a two-sample MR method, with the validation based on the NHANES database. Therefore, appropriate intervention in the timing of first sexual intercourse can reduce the risk of BC. However, AFS exhibits associations with other reproductive factors that may act together in BC progression. Further research is required to clarify the role of AFS in BC.

## Acknowledgments

The tables and figures in this review were created with WPS and R. In addition, we thank for Mr. Yao guidance for the usage of the NHANES database.

## Author contributions

**Conceptualization:** Min Ma.

**Data curation:** Xinghuang Yang.

**Formal analysis:** Xinghuang Yang.

**Funding acquisition:** Min Ma.

**Investigation:** Xinghuang Yang, Tianyu Deng, Tianai Xu.

**Methodology:** Xinghuang Yang, Min Ma.

**Project administration:** Min Ma.

**Resources:** Min Ma.

**Supervision:** Xinghuang Yang.

**Validation:** Xinghuang Yang, Tianyu Deng, Tianai Xu.

**Writing – original draft:** Xinghuang Yang.

**Writing – review & editing:** Xinghuang Yang, Tianyu Deng, Tianai Xu, Min Ma.

## Supplementary Material


