# Supplementary material for: Impact of age at first sexual intercourse on the development and prognosis of breast cancer: A two-sample Mendelian randomization study with NHANES validation
Source: Medicine (Baltimore). 2025 Aug 1;104(31):e43676. doi: 10.1097/MD.0000000000043676 (PMC12323919; doi:10.1097/MD.0000000000043676)
Supplement: Supplementary file 1 [file medi-104-e43676-s001.docx]

**Table S1** Summary of GWAS data included in this two-sample MR study

|  | PMID | Year | Author | Trait | GWAS ID | Sample size | Number of SNPs | Population |
| --- | --- | --- | --- | --- | --- | --- | --- | --- |
| Age at first sexual intercourse | 34211149 | 2021 | Mills MC | Age at first sexual intercourse | ebi-a-GCST90000047 | 397338 | 16359424 | European |
| Breast cancer | 34594039 | 2021 | Sakaue S | Breast cancer | ebi-a-GCST90018799 | 257730 | 24133589 | European |

**Table S2** Summary tables with palindromic sequences or incompatible SNPs

| SNPs | POS | CHR | EA | OA | β | SE | P |
| --- | --- | --- | --- | --- | --- | --- | --- |
| rs10922907 | 91193049 | 1 | T | A | 0.0211 | 0.0021 | 2.10E-24 |
| rs7525548 | 75001474 | 1 | T | A | 0.0145 | 0.0021 | 2.90E-12 |
| rs6747099 | 60777498 | 2 | C | G | 0.0123 | 0.0021 | 4.90E-09 |
| rs6744794 | 44842145 | 2 | G | C | -0.0191 | 0.0021 | 1.30E-19 |
| rs1226414 | 157109930 | 2 | T | A | 0.0144 | 0.0021 | 2.30E-12 |
| rs6748341 | 225377574 | 2 | G | C | 0.0149 | 0.0022 | 1.10E-11 |
| rs67723420 | 35775115 | 3 | A | T | 0.0146 | 0.0021 | 1.30E-11 |
| rs9835772 | 85766025 | 3 | T | A | -0.0138 | 0.0024 | 9.90E-09 |
| rs4602427 | 117474457 | 3 | G | C | -0.0172 | 0.0026 | 2.30E-11 |
| rs702 | 28710551 | 4 | T | A | 0.0181 | 0.0028 | 2.80E-10 |
| rs7381195 | 60030791 | 5 | A | T | -0.0136 | 0.0021 | 1.60E-10 |
| rs12653396 | 87847273 | 5 | A | T | -0.0199 | 0.0021 | 2.00E-21 |
| rs12523398 | 45119647 | 5 | A | T | 0.0219 | 0.0027 | 1.10E-15 |
| rs13178956 | 154786457 | 5 | T | A | -0.0164 | 0.0024 | 4.20E-12 |
| rs435538 | 24921398 | 5 | G | C | -0.017 | 0.0024 | 2.50E-12 |
| rs9403187 | 100324813 | 6 | T | A | -0.0134 | 0.0022 | 1.70E-11 |
| rs4728298 | 133436768 | 7 | A | T | -0.0219 | 0.0029 | 2.10E-14 |
| rs7815125 | 87680112 | 8 | A | T | 0.0179 | 0.0027 | 3.30E-11 |
| rs1585634 | 54396376 | 8 | C | G | -0.0146 | 0.0026 | 1.30E-08 |
| rs13280592 | 116686752 | 8 | G | C | -0.0134 | 0.0023 | 9.30E-09 |
| rs1991651 | 10706411 | 8 | G | C | 0.0182 | 0.0021 | 1.70E-17 |
| rs1547351 | 36842153 | 8 | A | T | 0.0123 | 0.0021 | 2.00E-08 |
| rs3447 | 86327243 | 9 | G | C | 0.0177 | 0.0029 | 3.70E-09 |
| rs2176337 | 108959330 | 9 | T | A | -0.0151 | 0.0022 | 1.10E-11 |
| rs4961705 | 16347927 | 9 | C | G | 0.013 | 0.0022 | 4.20E-09 |
| rs4246175 | 134930808 | 9 | A | T | 0.0144 | 0.0022 | 4.20E-11 |
| rs10749233 | 118777998 | 10 | C | G | 0.0175 | 0.0024 | 9.20E-13 |
| rs7942078 | 28656064 | 11 | T | A | 0.0161 | 0.0022 | 6.60E-14 |
| rs590414 | 105746052 | 11 | T | A | 0.0135 | 0.0021 | 1.70E-10 |
| rs7955865 | 56468706 | 12 | T | A | -0.0134 | 0.0022 | 8.40E-10 |
| rs1995181 | 24195048 | 12 | A | T | -0.0121 | 0.0021 | 4.50E-09 |
| rs9581878 | 28104552 | 13 | A | T | 0.0199 | 0.003 | 1.50E-10 |
| rs9514600 | 107644422 | 13 | G | C | -0.0114 | 0.0021 | 2.40E-08 |
| rs74737734 | 30726670 | 14 | T | A | 0.0353 | 0.0064 | 2.70E-08 |
| rs6564268 | 75606878 | 16 | G | C | 0.0266 | 0.0045 | 5.50E-09 |
| rs11866420 | 90054704 | 16 | G | C | -0.0168 | 0.0021 | 7.60E-16 |
| rs10469020 | 50811573 | 18 | T | A | 0.0217 | 0.0038 | 1.50E-08 |
| rs6058613 | 30864279 | 20 | G | C | -0.0171 | 0.0028 | 1.80E-09 |
| rs62177795 | 63475640 | 2 | A | G | 0.019 | 0.0025 | 9.00E-15 |

Note: POS:position; CHR:Chromosome; EA: Effector allele: OA: Non-effector allele; β: Allelic effect value; SE: Standard error of β

**Table S3** Results of MR analysis by five statistical methods

| Exposure | Method | Outcome | Beta | SE | P | OR(95%CI) |
| --- | --- | --- | --- | --- | --- | --- |
|  | MR Egger |  | -0.0769 | 0.3107 | 0.8048 | 0.9260  (0.5036 ~1.7024) |
|  | Weighted median |  | 0.1714 | 0.0936 | 0.0671 | 1.1870  (0.9880 ~1.4259) |
| Age at first sexual intercourse | Inverse variance weighted | Breast cancer | 0.2124 | 0.0707 | 0.0027 | 1.2366  (1.0766 ~1.4205) |
|  | Simple mode |  | 0.0809 | 0.2911 | 0.7815 | 1.0842  (0.5862 ~2.0053) |
|  | Weighted mode |  | -0.0540 | 0.3089 | 0.8615 | 0.9475  (0.5304 ~1.6926) |

**Table S4** Results of the tests for horizontal pleiotropy and heterogeneity

| Outcome | Exposure | Heterogeneity |  |  |  | Pleiotropy |  |
| --- | --- | --- | --- | --- | --- | --- | --- |
| BC | AFS | Q  (IVW) | P  value | Q  (MR-Egger) | P  value | MR-Egger  Intercept | P  value |
|  |  | 215.2953 | 0.0014 | 214.0406 | 0.0014 | 0.0046 | 0.3404 |

**Table S5** Baseline characteristics of participants with BC in NHANES 1999-2016

| Characteristic |  | Survival state | | P |
| --- | --- | --- | --- | --- |
|  | Total | survival | death |  |
| Patients, n | 207 | 184 | 23 |  |
| Follow-up time, years | 9.12 ± 4.66 | 9.51 ± 4.60 | 5.96 ± 3.88 | 0.001 |
| Age, years | 56.38 ± 8.39 | 56.34 ± 8.61 | 56.70 ± 6.55 | 0.9 |
| Race, n(%) |  |  |  | 0.05 |
| Non-Hispanic White | 97.00 (46.86%) | 92.00 (50.00%) | 5.00 (21.74%) |  |
| Non-Hispanic Black | 49.00 (23.67%) | 41.00 (22.28%) | 8.00 (34.78%) |  |
| Mexican American | 22.00 (10.63%) | 19.00 (10.33%) | 3.00 (13.04%) |  |
| Other | 39.00 (18.84%) | 32.00 (17.39%) | 7.00 (30.43%) |  |
| Education level, n(%) |  |  |  | 0.003 |
| Less than high | 30.00 (14.49%) | 22.00 (11.96%) | 8.00 (34.78%) |  |
| High school grad or equivalent | 38.00 (18.36%) | 32.00 (17.39%) | 6.00 (26.09%) |  |
| College or above | 139.00 (67.15%) | 130.00 (70.65%) | 9.00 (39.13%) |  |
| Family income-poverty ratio, n(%) |  |  |  | 0.029 |
| ≤1.30 | 50.00 (24.15%) | 40.00 (21.74%) | 10.00 (43.48%) |  |
| 1.3-3.5 | 54.00 (26.09%) | 47.00 (25.54%) | 7.00 (30.43%) |  |
| >3.5 | 103.00 (49.76%) | 97.00 (52.72%) | 6.00 (26.09%) |  |
| BMI, Kg/m2 |  |  |  | 0.2 |
| < 25.0 | 58.00 (28.02%) | 51.00 (27.72%) | 7.00 (30.43%) |  |
| 25.0-29.9 | 50.00 (24.15%) | 48.00 (26.09%) | 2.00 (8.70%) |  |
| ≥30 | 99.00 (47.83%) | 85.00 (46.20%) | 14.00 (60.87%) |  |
| Smoking status, n(%) |  |  |  | 0.005 |
| Never | 119.00 (57.49%) | 107.00 (58.15%) | 12.00 (52.17%) |  |
| Former | 61.00 (29.47%) | 58.00 (31.52%) | 3.00 (13.04%) |  |
| Current | 27.00 (13.04%) | 19.00 (10.33%) | 8.00 (34.78%) |  |
| Drink status, n(%) |  |  |  | 0.08 |
| Never | 151.00 (72.95%) | 130.00 (70.65%) | 21.00 (91.30%) |  |
| Moder | 24.00 (11.59%) | 24.00 (13.04%) | 0.00 (0.00%) |  |
| Heavy | 32.00 (15.46%) | 30.00 (16.30%) | 2.00 (8.70%) |  |
| Hypertension, n(%) | 120.00 (57.97%) | 105.00 (57.07%) | 15.00 (65.22%) | 0.5 |
| Diabetes, n(%) | 32.00 (15.46%) | 24.00 (13.04%) | 8.00 (34.78%) | 0.012 |
| eGFR(mL/min/1.73m^2^) | 89.96 ± 16.76 | 90.15 ± 16.91 | 88.42 ± 15.74 | 0.6 |
| Age of first sexual intercourse,year | 18.91 ± 4.65 | 18.74 ± 4.02 | 20.26 ± 8.14 | >0.9 |

**Table S6** Subgroup analysis of AFS and all-cause mortality of patients with BC

| Character | HR(95%CI) | P | P for interaction |
| --- | --- | --- | --- |
| Race |  |  | 0.092 |
| Non-Hispanic White | 1.27(1.17-1.379) | <0.001 |  |
| Other | 1.049(0.92-1.196) | 0.472 |  |
| Hypertension |  |  | 0.194 |
| No | 1.203(1.104-1.311) | <0.001 |  |
| Yes | 0.958(0.809-1.135) | 0.619 |  |
| Diabetes |  |  | 0.604 |
| No | 1.154(1.038-1.283) | 0.008 |  |
| Yes | 1.257(1.046-1.51) | 0.015 |  |
| Age of first intercourse |  |  | - |
| <18 | 0.795(0.514-1.229) | 0.302 |  |
| >=18 | 1.175(1.079-1.281) | <0.001 |  |
