# Supplementary material for: Impact of age at first sexual intercourse on the development and prognosis of breast cancer: A two-sample Mendelian randomization study with NHANES validation
Source: Medicine (Baltimore). 2025 Aug 1;104(31):e43676. doi: 10.1097/MD.0000000000043676 (PMC12323919; doi:10.1097/MD.0000000000043676)

**Figure S1** The Forest plot of the causal association between AFS and BC


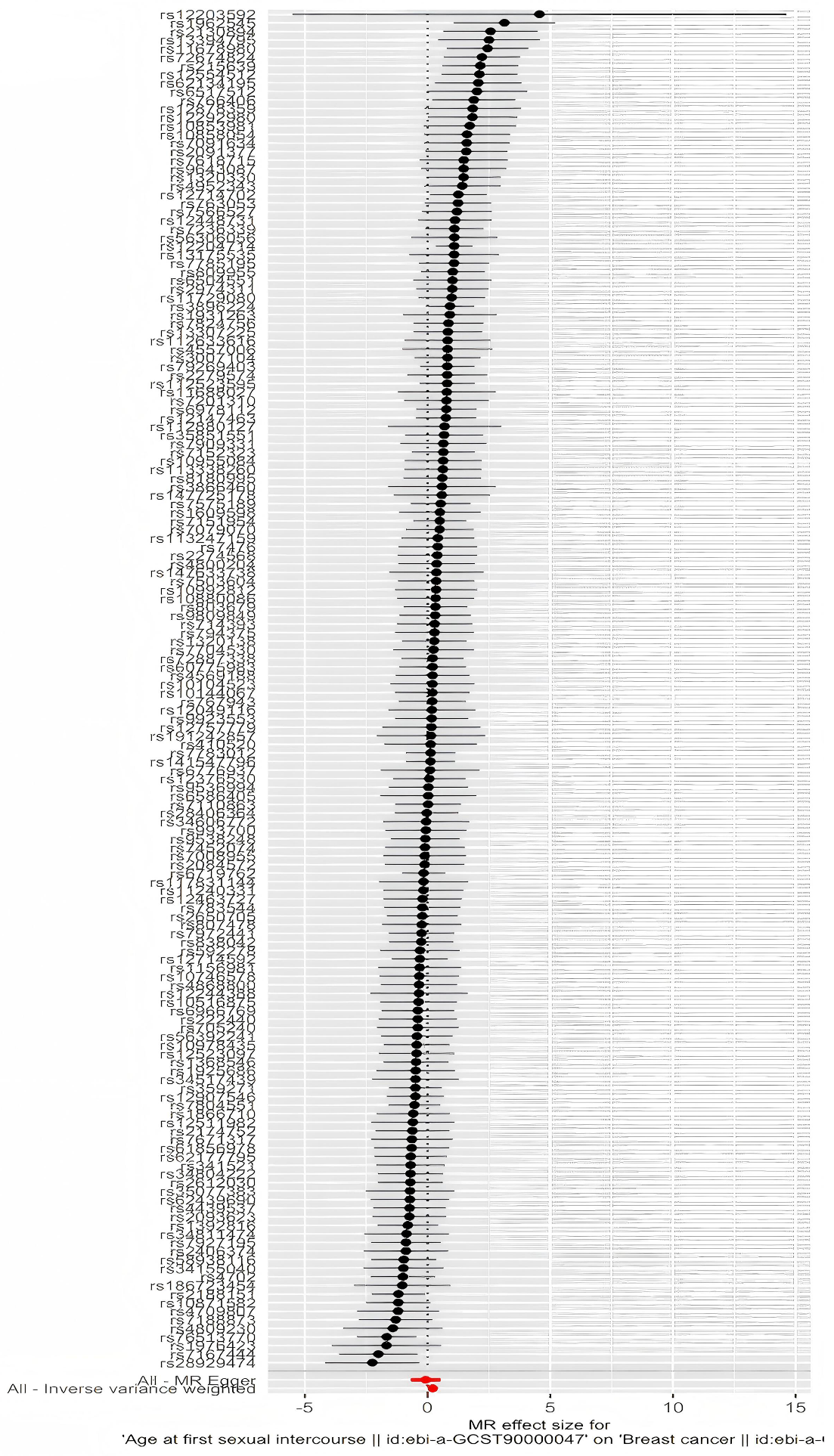


**Figure S2** The Funnel plot of the causal association between AFS and BC


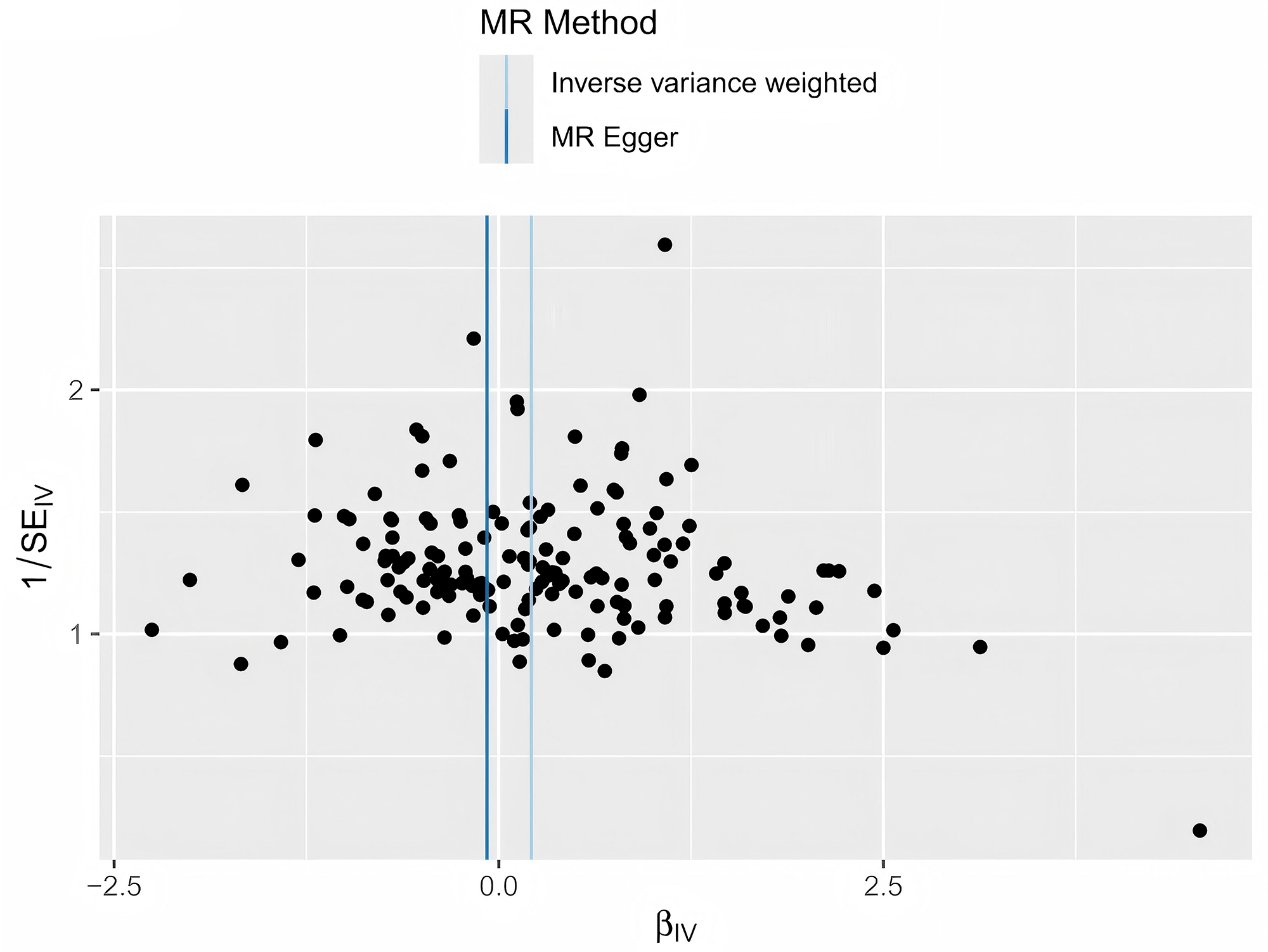


**Figure S3** The Leave-one-out test of the causal association between AFS and BC


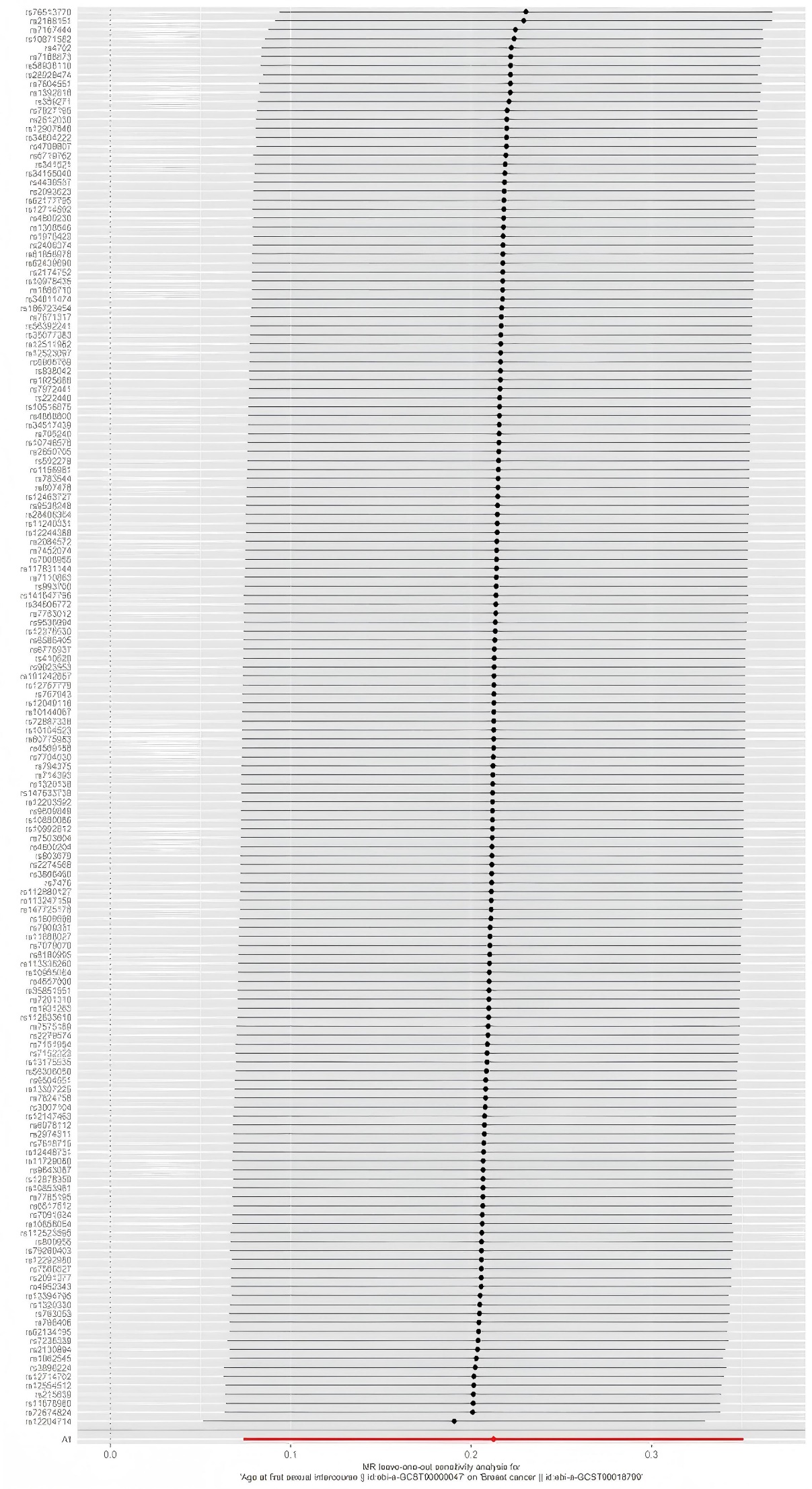


**Figure S4** The Kaplan-Meier(KM) curves analysis


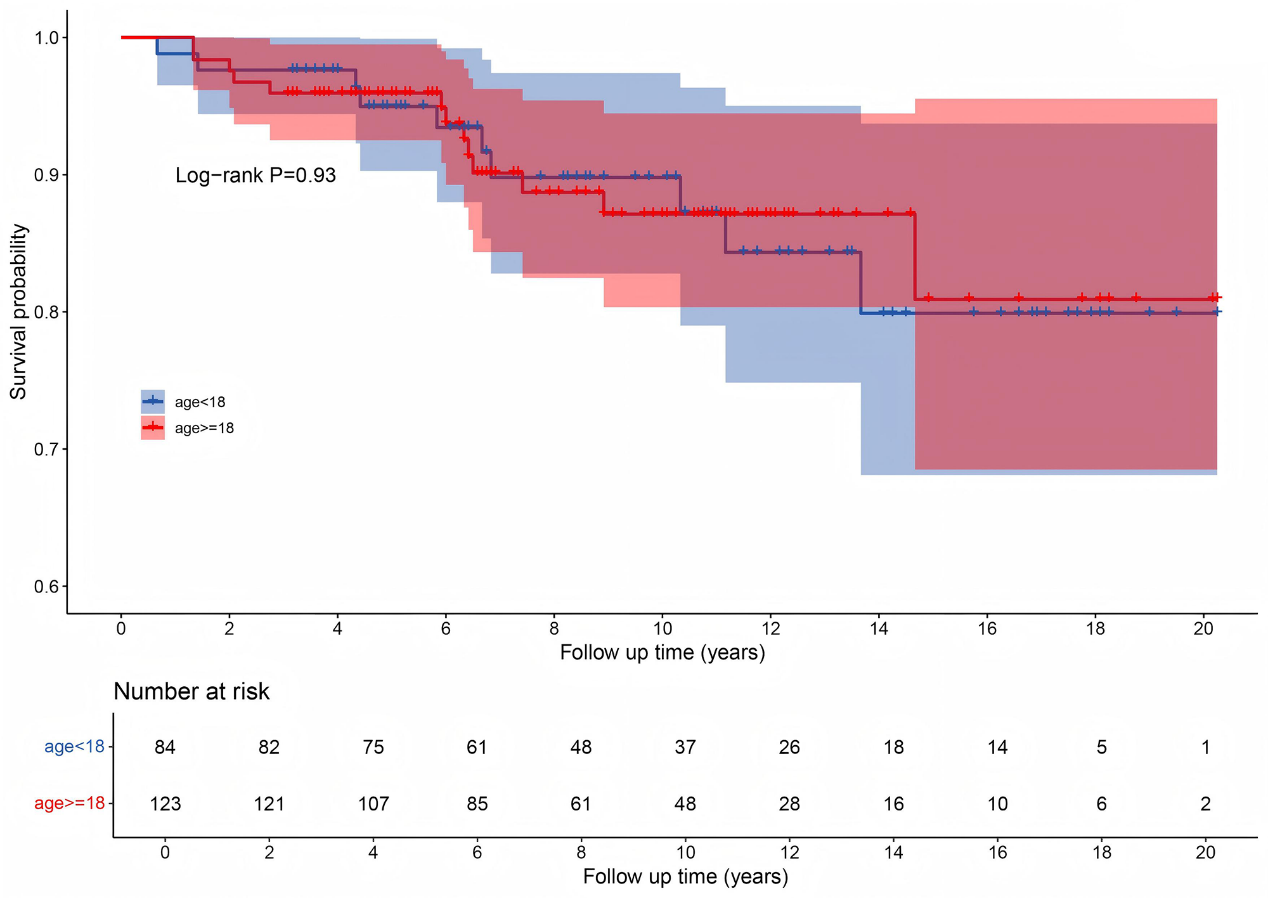


**Figure S5** The restricted cubic splines(RCS) curves ayalysis


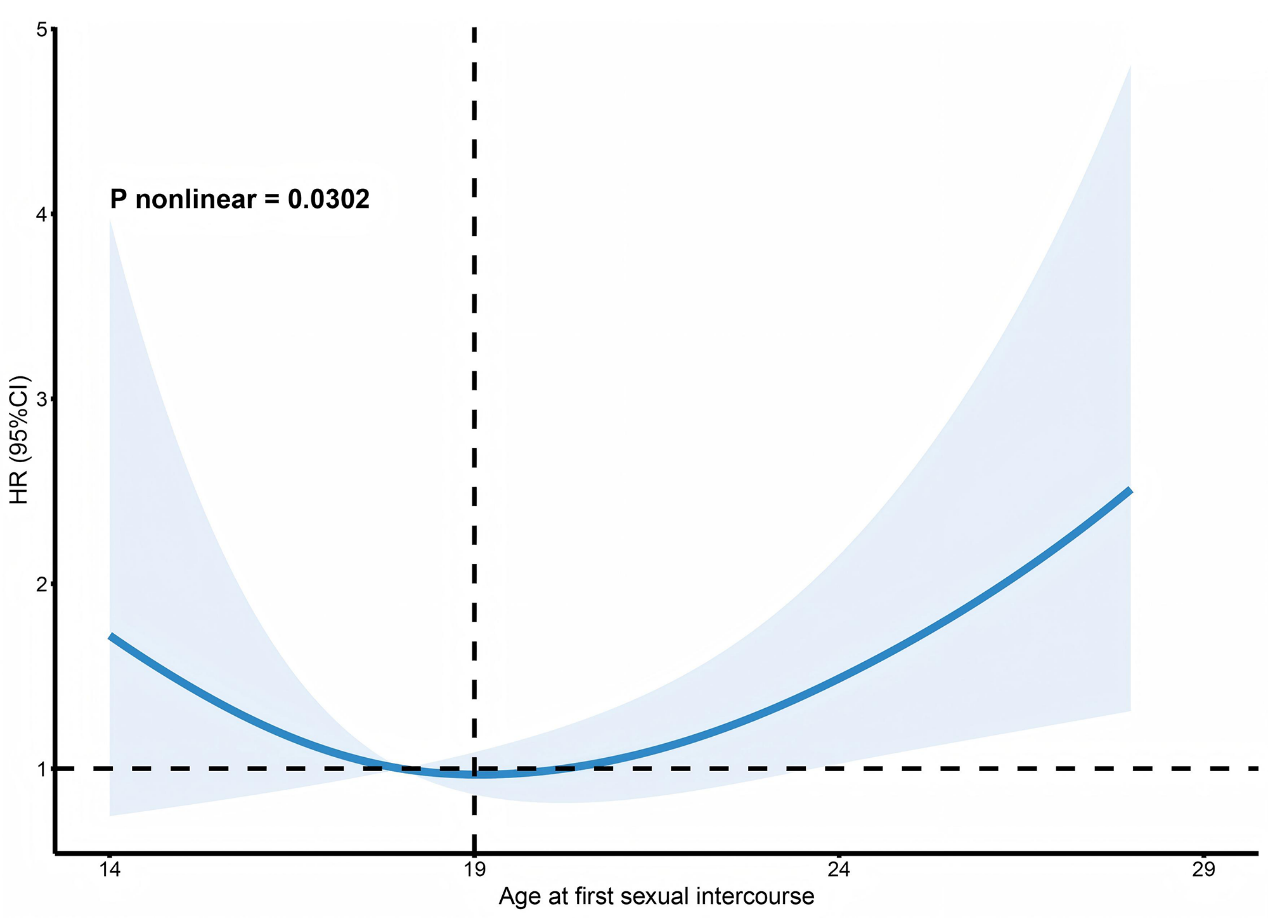

Supplement: Supplementary file 2 [file medi-104-e43676-s002.docx]
